# Substance use in patients with eating disorders - a review of the current evidence

**DOI:** 10.1192/j.eurpsy.2023.1612

**Published:** 2023-07-19

**Authors:** T. P. V. Alves, P. Nunes, S. Timóteo

**Affiliations:** 1 Department of Psychiatry, Centro Hospitalar do Médio Tejo, Tomar; 2 Department of Psychiatry, Centro Hospitalar Universitário de São João, Porto, Portugal

## Abstract

**Introduction:**

Eating disorders are potentially severe, complex, and life-threatening. Therefore, it is crucial to identify and treat all the comorbidities that could worsen the prognosis.

**Objectives:**

The aims of this work are to assess if substance use disorders are frequently comorbid in patients suffering from eating disorders, what are the problems associated with drug consumption among these individuals, and what are the best therapeutic strategies in this dual psychiatric diagnosis.

**Methods:**

We carried out a narrative review, by searching on PubMed and Google Scholar databases, using the expression “eating disorders and substance use disorders”. We included all types of scientific articles published between 2018 and 2022, and information was extracted regarding the objectives of this review.

**Results:**

The prevalence rates of substance use in eating disorders are higher than in general population. For eating disorders in general, substance use disorder (SUD) is the third most prevalent psychiatric comorbidity. According to a meta-analysis published in 2019, the lifetime prevalence rate of a comorbid SUD was 21.9% (95% CI 16.7-28.0). SUDs were more frequently comorbid among individuals with the binge/purge type, which has a specific phenotype characterized by greater impulsivity, emotional dysregulation, and problems with executive control. Individuals with this dual diagnosis had a higher number of psychiatric comorbidities, were more likely to be prescribed mood stabilizers, and were more sensitive to reward.

Research suggests that eating disorders patients with co-occurring SUDs experience lower rates of treatment response, higher relapse rates, more severe medical complications, greater impairment, poorer long-term outcome, and are at higher risk of early mortality.

To date, there is limited information guiding the concurrent treatment of eating disorders and SUDs. Dialectical Behavior Therapy for SUDs seems to be a promising treatment, but more research on its efficacy will be essential for establishing treatment protocols for these patients.

**Conclusions:**

SUDs have an impact on treatment response and increase mortality among people with eating disorders. The prevention and treatment of SUDs in this patient group is thus imperative to reduce mortality and increase the quality of life of these patients.

**Disclosure of Interest:**

None Declared

